# Pure small cell neuroendocrine carcinoma of urinary bladder: A case report

**DOI:** 10.1002/ccr3.6156

**Published:** 2022-08-24

**Authors:** Abbas Eshraghi, Mohammad Mehdi Riyahi, Afshin Ghaderi, Maedeh Alsadat Fatemi, Azhar Eshraghi, Danial Fazilat‐Panah

**Affiliations:** ^1^ Department of Internal Medicine, Hematology & Oncology, School of Medicine Qom University of Medical Sciences Qom Iran; ^2^ Clinical Research Development Qom University of Medical Sciences Qom Iran; ^3^ Department of Internal Medicine, Hematology & Oncology, School of Medicine Yasuj University of Medical Sciences Yasuj Iran; ^4^ Cancer Research Center Babol University of Medical Sciences Babol Iran; ^5^ Qom University of Medical Sciences Qom Iran

**Keywords:** bladder tumor, chromogranin, small cell neuroendocrine carcinoma, synaptophysin

## Abstract

Small cell neuroendocrine carcinoma of the bladder is rare. We report a case of small cell carcinoma of the bladder with extensive regional lymph node metastases who underwent radical cystoprostatectomy and subsequent adjuvant systemic chemotherapy and chemoradiotherapy.

## Graphical abstract

In patients with high‐grade poorly differentiated neoplasms of bladder, small cell neuroendocrine carcinoma should be ruled‐out through comprehensive immunohistochemistry evaluation considering the different work‐up approach and treatment regimens.

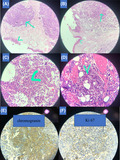

## 1 INTRODUCTION

Small cell neuroendocrine carcinoma of the bladder is a rare human cancer consisting less than 1.5% of all nonurothelial cell carcinoma of the bladder. Cramer et al. described it in the first instance in the 1990s,^1^ however, the evidence is limited to case reports, case series or cohort studies until now.^2^, ^3^

Small cell neuroendocrine carcinoma of the bladder occurs more commonly in men during the 6th and the 7th decades of life.^4^, ^5^ Unlike transitional cell carcinoma, it associates with a worse prognosis and more aggressive behaviors.^5^, ^6^ The median survival with and without treatment were reported 12–24 months and 4–5 months, respectively.^7^ The prognosis is generally poor because of the aggressive nature of disease specially in most undifferentiated huge tumors and up to 80% of patients have distant metastasis at diagnosis.^8^

In the current report, a case of small cell carcinoma of the bladder with extensive regional lymph node metastases who underwent radical cystoprostatectomy and subsequent adjuvant systemic chemotherapy and chemoradiotherapy was reported.

## 2 CASE PRESENTATION

A 50‐year‐old man without any documented past medical history was admitted to the department of urology because of the presence of blood in his urine (i.e., gross hematuria). The smoking was the only known risk factor in the patient. The gross hematuria had begun from 3 months ago and continued intermittently. There was no history of dysuria, nocturia, urinary frequency, or urinary incontinence. The physical examination showed a round painless non tender mass which was located at the hypogastric region. At the beginning, the complete blood count, the biochemical profile, and urine analysis were requested which were normal except too many red blood cells detected in the urine analysis.

**FIGURE 1:**
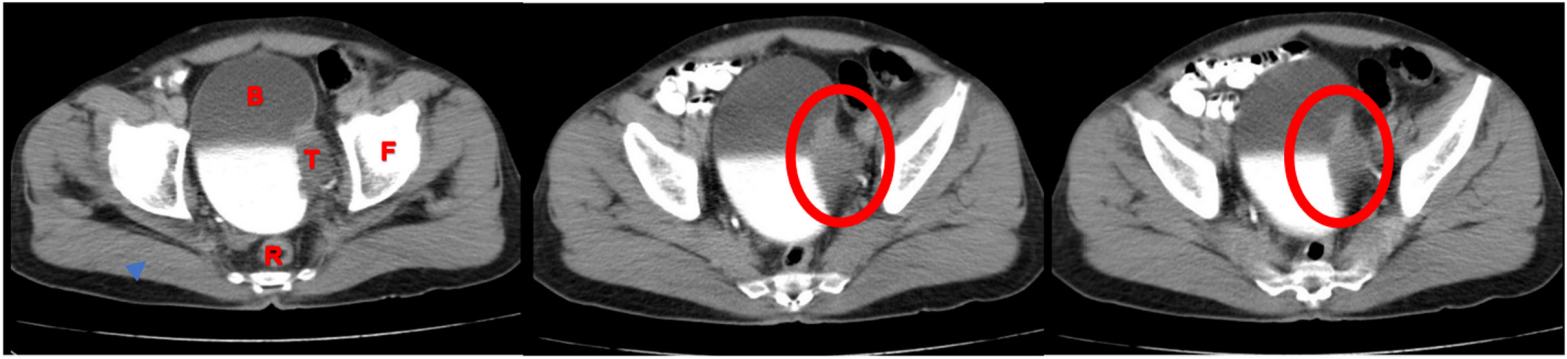
CT scan of pelvis shows an intravesical mass (red ellipse and T word indicate the tumoral mass) on the left lateral border of the bladder (B: bladder, T: tumoral mass, R: rectum, F: femur head)

Ultrasound imaging of the abdomen revealed a locally advanced 5 × 3 cm bladder mass involving its dome and left lateral and anterior walls. Further assessments by intravenous contrast abdominopelvic computed tomography (CT) scan and CT urogram showed bladder wall thickening and polypoid filling defect arising the bladder dome and its left lateral and anterior walls measuring 5 × 3 cm without any evidence of extra‐vesical invasion (Figure 1). Also, multiple lymph node metastases were detected in the common iliac lymph nodal regions. The direct visual examination via cystoscopic evaluation showed a massive hemorrhagic lobular mass at the bladder dome with extension toward the left lateral and anterior walls. A cystoscopic biopsy showed a high‐grade poorly differentiated neoplasm. A chest CT scan and whole‐body bone scan were obtained to rule out potential lung and bone metastases, respectively. Both investigations were reported no further tumoral involvement.

**FIGURE 2:**
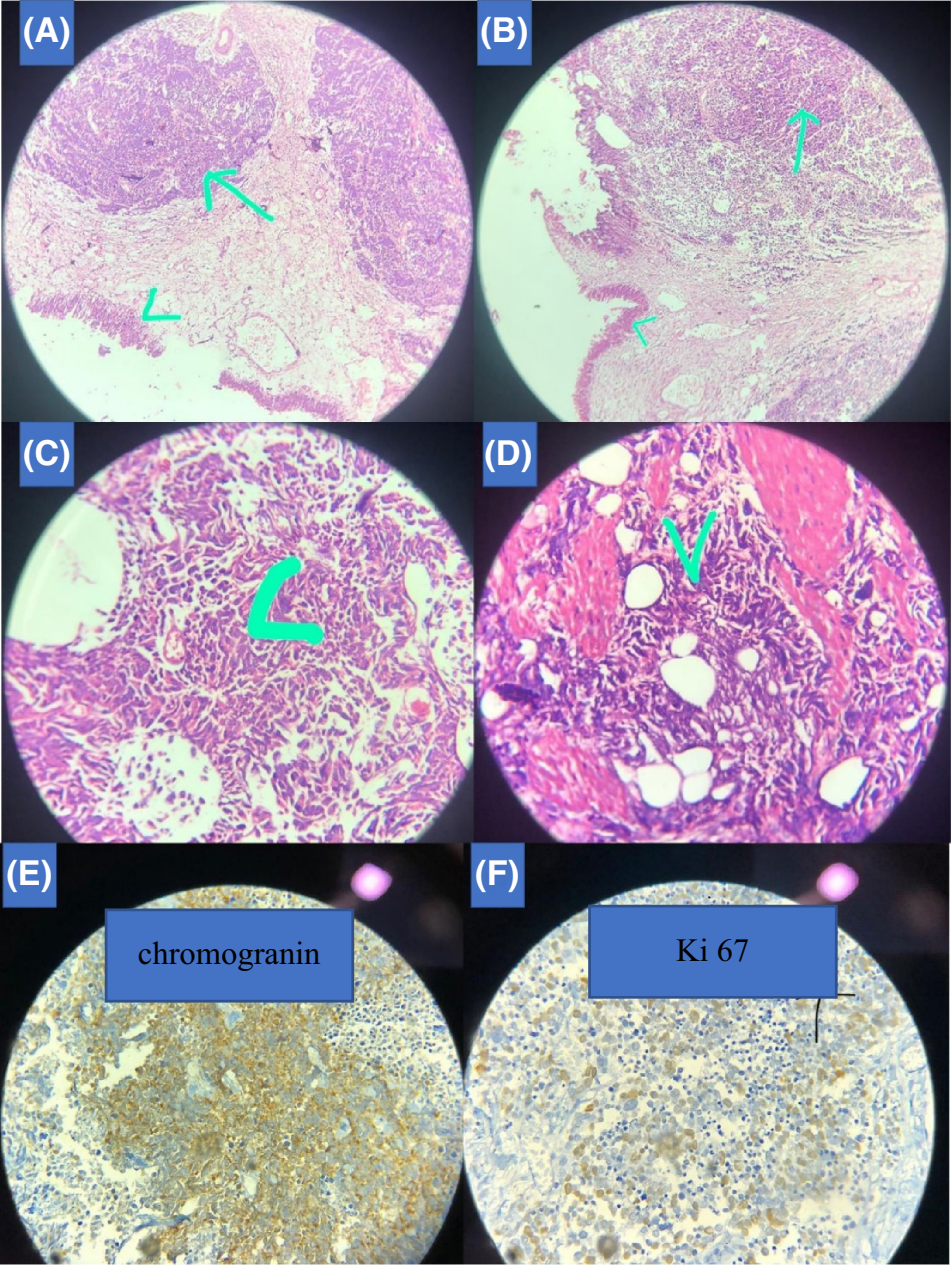
Light microscopic and immunohistochemical evaluation of tumoral lesion. Microscopic evaluation of bladder shows a malignant neoplasm of atypical medium sized cells with irregular nuclei, fine chromatin, scant cytoplasm, and high mitotic activity (A–D). In Figure A–D, the tip of arrow shows the tumoral lesion and the tip of arrowhead shows the normal epithelium [A and B: ×100 and C and D: ×250]. IHC studies showed a positive staining for chromogranin (100% of tumoral cells) (E) and high expression of Ki67 (80% of tumoral cells) (F)

Subsequently, the patient underwent a radical cystoprostatectomy with bilateral pelvic lymphadenectomy. There was a unifocal 5 × 3 × 2 cm ulcerated tumoral lesion located at the dome and the left lateral and anterior walls of bladder. Microscopic examination showed a poorly differentiated small‐cell‐type neuroendocrine tumors which invades through the muscularis propria into perivesical tissue, microscopically. One out of five resected lymph nodes on the right iliac lymphatic region and two out of three resected lymph nodes on the left iliac lymphatic region were involved by tumor. Left ureter margin and external surface of perivesical soft tissue were infiltrated by tumoral cells. The final pathologic stage was R1 resection with pT3aN2M0 based on the AJCC cancer staging system, 8th edition (Figure 2). Immunohistochemical staining revealed a TTF‐1, CK, chromogranin, and synaptophysin existing predominantly in the tumoral cells. It also showed that there was no expression of CK5/6, GATA3, HMB45, and CDX2. Ki‐67 expression was more than 80% (Figure 2).

After surgery, four cycles of adjuvant cisplatin [80 mg/m^2^ given intravenously on Day 1] and etoposide [100 mg/m^2^ intravenously on Days 1–3], every 3–4 weeks were prescribed. Subsequently, adjuvant chemoradiation was considered which consisted of 30 fractions of 200 (centigray) cGy per fraction (total dose = 60 Gy) with concurrent weekly cisplatin [40 mg/m^2^ given intravenously].

After completion of adjuvant treatments, patient was followed up every 3 months by physical examination and history taking. Also, a whole‐body CT scan was obtained every 6 months. With a follow‐up of 12 months, the patient was disease free.

## 3 DISCUSSION

In this manuscript, we report a man with advanced stage small cell carcinoma of the bladder who underwent radical cystoprostatectomy and adjuvant systemic chemotherapy and chemoradiotherapy who survived for at least 12 months after completion of his treatments. Neuroendocrine tumors are mostly seen in the gastrointestinal track, prominently in pancreas, small intestine, and rectum.^9^ The prognosis of patients largely depends on the location of tumor, its grade, its size and stage nodal metastases, and Ki67 expression level.^10^, ^11^, ^12^

But as mentioned earlier, the small cell carcinoma of the bladder is a rare entity. Koay et al. (2022), in one of the largest series on the patients with small cell carcinoma of the bladder, reviewed the Surveillance, Epidemiology, and End Results (SEER) database and found 642 patients from 1991 to 2005.^4^ Overall incidence was from 3‐fold higher in men and most of cases were diagnosed with a locally advanced disease at the presentation. Transurethral resection of the bladder tumor without any further adjuvant treatment was the most common surgical treatment resulting in a median survival of 12 months. Another study by Royce et al (2017) assessed the data of all of nonurothelial cell carcinoma of the bladder from 1998 to 2014 in the United States using the National Cancer Data Base.^5^ Out of 10,421 patients with nonurothelial cell carcinoma of the bladder, 1.3% suffered from neuroendocrine tumors which was more frequent in men and was diagnosed at the higher stages. Their data showed that surgery without adjuvant treatment was the main treatment pattern, although the prognosis was dismal.

In the presented patient, immunohistochemical examinations showed that tumoral cells were stained by TTF‐1, CK, chromogranin, and synaptophysin. Abrahams et al. (2005) in a study of 51 patients with small cell carcinoma of the bladder reported that synaptophysin and chromogranin were positive in 30%–70% of the cases.^7^ Both of these immunohistochemical tumor markers are among the main characteristic indicators of neuroendocrine tumors and their presence confirm the diagnosis.^13^ Moreover, unlike transitional cell carcinoma, TTF‐1 positivity was reported in almost half of cases with small cell carcinoma of the urinary bladder in a study of 44 patients by Jones et al. (2005).^14^, ^15^

Neuroendocrine tumors are rare and can occur anywhere in the body. Nevertheless, in any high‐grade undifferentiated tumors, the presence of neuroendocrine tumors should be ruled out properly due to their unique nature and their exceptional prognosis. In this context, immunohistochemical studies have a prominent role that can provide evidence of the neuroendocrine origin.^16^

The new radiotherapy techniques provide this opportunity to precisely deliver higher doses to the tumor bed and to effectively spare the normal tissues leading to the better tumor control especially in patients with surgical positive margins such as the presented case.^17^, ^18^ Besides, there are promising new data on the underlying molecular mechanisms of different cancers that may improve the outcome of patients with malignant diseases through introducing new drugs and treatment modalities.^19^, ^20^, ^21^, ^22^, ^23^, ^24^

## 4 CONCLUSION

In patients with high‐grade poorly differentiated neoplasms of bladder, small cell neuroendocrine carcinoma should be considered by oncologists and pathologist, particularly in elderly men. The diagnosis should be ruled out through comprehensive immunohistochemical examination, which includes chromogranin and synaptophysin.

## AUTHOR CONTRIBUTIONS

All authors contributed equally.

## CONFLICT OF INTEREST

The authors declare that they have no conflicts of interest.

## ETHICAL APPROVAL

The study was approved by the Ethical Committee of Qom University of Medical Sciences.

## CONSENT

Written informed consent was obtained from the patient to publish this report in accordance with the journal's patient consent policy.

## Data Availability

The data sets used and/or analyzed during the current study are available from the corresponding authors per request.
